# CircNFIX regulates chondrogenesis and cartilage homeostasis by targeting the miR758‐3p/KDM6A axis

**DOI:** 10.1111/cpr.13302

**Published:** 2022-07-05

**Authors:** Hongyi Liao, Qingqiang Tu, Yunze Kang, Guping Mao, Zhiwen Li, Shu Hu, Puyi Sheng, Xudong Wang, Yiyang Xu, Dianbo Long, Yuanyuan Xu, Yan Kang, Ziji Zhang

**Affiliations:** ^1^ Department of Joint Surgery, The First Affiliated Hospital Sun Yat‐sen University Guangzhou China; ^2^ Guangdong Provincial Key Laboratory of Orthopaedics and Traumatology, The First Affiliated Hospital Sun Yat‐sen University Guangzhou China; ^3^ Department of Joint Surgery, Center for Orthopaedic Surgery The Third Affiliated Hospital of Southern Medical University Guangzhou China; ^4^ Department of Pediatric Nephrology and Rheumatology, The First Affiliated Hospital Sun Yat‐sen University Guangzhou China

## Abstract

**Objectives:**

Osteoarthritis (OA) is a degenerative disease causing the progressive destruction of articular cartilage; however, the aetiology has not yet been elucidated. Circular RNAs (circRNAs) are reportedly involved in cartilage degeneration and OA development. In the present study, we identified that circNFIX regulates chondrogenesis and cartilage homeostasis.

**Materials and Methods:**

Microarray analysis was performed to explore circRNA expression during the chondrogenic differentiation of human adipose‐drived stem cells (hADSCs). CircNFIX expression was determined using quantitative reverse transcription‐polymerase chain reaction and in situ hybridization. Gain‐ and loss‐of‐function assays were performed to validate the role of circNFIX in cartilage homeostasis. RNA pull‐down, Argonaute2‐RNA immunoprecipitation and luciferase reporter assays were performed to evaluate the interactions among circNFIX, miR758‐3p and KDM6A.

**Results:**

CircNFIX expression was upregulated in the early and middle stages, whereas downregulated in the late stage of hADSC chondrogenesis. CircNFIX inhibition attenuated hADSC chondrogenesis. CircNFIX was remarkably downregulated in OA samples, circNFIX overexpression protected against chondrocyte degradation and alleviated OA progression in the destabilization of the medial meniscus OA model. Mechanistically, circNFIX acted as a sponge of miR758‐3p and played a role in the chondrogenesis and chondrocyte degeneration by targeting the miR‐758‐3p/KDM6A axis.

**Conclusions:**

Our results revealed a key role of circNFIX in chondrogenesis and cartilage homeostasis, which may provide a potential therapeutic strategy for OA treatment.

## Graphical abstract

Schematic illustration of the effects of circNFIX on human adipose‐drived stem cells (hADSCs) chondrogenesis and cartilage homeostasis. The results of this study revealed that circNFIX can promote the chondrogenic differentiation of hADSCs and maintain cartilage homeostasis. Mechanistically, circNFIX acted as a sponge of miR758‐3p regulated the methylation level of SOX9 by targeting the miR‐758‐3p/KDM6A axis, thereby mediating the chondrogenic differentiation of hADSCs and cartilage homeostasis. The circNFIX/miR‐758‐3p/KDM6A axis may serve as an effective therapeutic target for cartilage degeneration and OA treatment.

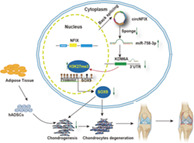

## 1 INTRODUCTION

Osteoarthritis (OA) is a degenerative disease whose incidence increases annually with the ageing of the world's population.^1^ It is characterized by the progressive destruction of articular cartilage, bone remodelling, osteophyte formation, joint inflammation and loss of normal joint functioning.^2^ Current clinical management of OA is limited to palliative drugs or joint replacement surgery with no effective biological agent for treating cartilage degeneration. In recent years, stem cell composite materials have been used for cartilage repair.^3^ Several studies have reported that human adipose‐drived stem cells (hADSCs) have the potential for multidirectional differentiation and can be used as seed cells for cartilage repair.^4^, ^5^, ^6^ Researchers have found that although tissue‐engineered cartilage can maintain good cartilage properties in the early stage, due to the inflammatory environment in the arthritic joint, the seed cell‐derived chondrocytes are prone to hypertrophic differentiation, and the cartilage in the repair area gradually degenerates in the late stage.^7^, ^8^ Hence, the fundamental problem of cartilage degeneration is yet to be solved.

Circular RNAs (circRNAs) are covalently closed, endogenous biomolecules in eukaryotes.^9^ circRNAs have been reportedly involved in various diseases, such as atherosclerosis and nervous system disorders.^10^, ^11^, ^12^ CircRNA_0001236, derived from mesenchymal stem cells (MSCs), mediates chondrogenesis and prevents cartilage degradation.^13^ Shen et al. reported that CircSERPINE2 alleviates OA progression by targeting microRNA (miR)‐1271 and ETS‐related gene.^14^ However, the role and mechanism of circRNA in chondrogenesis regulation and cartilage degradation remain unknown.

Recent studies have reported that some circRNAs can function as microRNA (miRNA) sponges and competitively inhibit miRNA activity.^15^ Multiple studies have reported miRNA involvement in chondrogenesis and cartilage degeneration. miR‐490‐5p attenuates hADSC chondrogenesis and chondrocyte homeostasis.^16^ miR‐204 and miR‐211 maintain joint homeostasis and prevent OA development.^17^ circRNAs may serve as central regulators of these miRNAs. CircSLC7A2 regulates chondrocyte catabolism and anabolism by sponging miR‐4498/TIMP3 and protecting against OA development.^18^ CircRNA.33186 contributes to OA pathogenesis by sponging miR‐127‐5p.^19^ Therefore, studying the regulatory network of circRNAs and miRNAs will help clarify the pathogenesis of cartilage degeneration.

Accordingly, the present study aimed to investigate the functions and molecular mechanisms of circRNAs in chondrogenesis and cartilage homeostasis. We identified a circRNA (has_circ_005660, termed circNFIX) that was upregulated during hADSC chondrogenesis and downregulated in OA and systematically explored its role in vitro and in vivo.

## 2 MATERIALS AND METHODS

### 2.1 hADSC culture and induction of chondrogenesis

This study was approved by the Clinical Research Ethics Committee ([2013] C‐260). hADSCs were obtained from three normal donors who underwent liposuction (age <35 years). The hADSCs were separated and cultured as previously described.^20^ Briefly, adipose tissue was collected in a centrifuge tube (50 ml) and washed twice with phosphate‐buffered solution (PBS). The adipose tissue was incubated in a 0.075% collagenase type I solution prepared in PBS containing 2% P/S and 1% penicillin–streptomycin (PS) for 30 min at 37°C, 5% CO_2_. After neutralizing the collagenase type I with 5 ml of α‐Modified Eagle Medium (α‐MEM) containing 20% foetal bovine serum (FBS), the sample was centrifuged at 2000 rpm for 5 min; the supernatant was removed, the pellet was washed twice with PBS and centrifuged at 2000 rpm for 5 min. Next, the supernatant was removed, and the sediment was resuspended in 3 ml of α‐MEM containing 20% FBS and then filtered through a 70 μm cell strainer. Finally, cells were plated in a 75 cm^2^ culture flask and cultured with α‐MEM containing 20% FBS and 1% PS in an incubator at 37°C with 5% CO_2_. Chondrogenesis was induced by micromass culture based on a previous protocol.^21^ hADSCs were collected and resuspended in a chondrogenic medium (Cyagen Biosciences, China) at 2 × 10^7^ cells/ml; 12.5 μl of the cell suspension was dropped into each well of a 24‐well plate; hADSCs were allowed to dry for 90 min at 37°C; then, 500 μl of chondrogenic medium were added to each well. The microspheres were cultured in a 24‐well plate with a chondrogenic medium and collected for experimental purposes at different time points (3, 7, 14, 21, 28 or 35 days).

### 2.2 Human cartilage collection and chondrocyte culture

Human cartilage samples were collected according to protocols approved by the Clinical Research Ethics Committee ([2021]334). Pathological cartilage tissues were harvested from OA‐affected knee joints during total knee replacement surgeries (*n* = 15). Normal cartilage tissues were collected from patients (*n* = 15) who underwent total hip replacement surgery with femoral neck fractures without having any history of OA or rheumatoid arthritis. Chondrocytes were isolated from human cartilage tissues as previously described.^16^ Briefly, the cartilage was separated from the subchondral bone, then washed twice with PBS; cartilage samples were mixed with 4 mg/ml protease prepared inDulbecco's Modified Eagle Medium/Nutrient Mixture F12 (DMEM/F12) containing 10% FBS (Gibco, Life Technology, MA, USA) and 1% PS. Cartilage specimens were incubated in a constant temperature shaker at 37°C, 90 rpm, for 90 min. Next, the supernatant was removed; cartilage samples were washed twice with PBS, mixed with 0.25 mg/ml collagenase P prepared in DMEM/F12 containing 10% FBS and 1% PS, then incubated in a constant temperature shaker at 37°C, 50 rpm, for 8 h. Finally, the supernatant was filtered through a 70 μm cell strainer. Chondrocytes were collected by centrifuging the supernatant, and cells were cultured in a 75 cm^2^ culture flask and maintained in DMEM containing 10% FBS and 1% PS. SW1353 and HEK293T cells were obtained from the American Type Culture Collection (Manassas, VA, USA) and cultured in DMEM with 10% FBS and 1% PS. All cells were grown in an incubator at 37°C with 5% CO_2_ (Thermo Fisher Scientific, USA).

### 2.3 RNA interference and overexpression

Primary human chondrocytes (PHCs) were transfected with pcDNA3‐circNFIX plasmids (Hanbio, Shanghai, China) using Lipofectamine R 3000 Transfection Reagent (Invitrogen, Carlsbad, USA). hADSCs and PHCs were transfected with small interfering RNA (siRNA) for circNFIX or KDM6A and miR‐758‐3p‐mimic or ‐inhibitor (RiboBio, Guangzhou, China) using the Lipofectamine RNAiMAX transfection reagent (Thermo Fisher Scientific, USA). After hADSC monolayers were transfected, hADSCs were collected, and chondrogenic differentiation was induced by micromass culture; 3 days later, the microspheres were transfected again. The sequences were as follows: circNFIX siRNA, 5′‐GTGACAGAGCTGGTGAGAGTA‐3′; miR‐758‐3p‐mimic, 5′‐UUUGUGACCCACUAACC‐3′, 5′‐GGUUAGUGGACC AGGUCACAAA‐3′; miR‐758‐3p‐inhibitor, 5′‐GGUUAGUGGACCAGGUCACA AA‐3′; KDM6A siRNA#1, 5′‐GGACUUGCAGCACGAAUUATT‐3′; KDM6A siRNA#2, 5′‐TTTATTCCTTAGTCTATGTGC‐3′.

### 2.4 RNA extraction and quantitative reverse transcription‐polymerase chain reaction (qRT‐PCR) analysis

Total RNA from the PHCs and hADSC chondrogenic microspheres were collected using TRIzol reagent (Invitrogen, Carlsbad, CA, USA). Reverse transcription was performed using Mir‐XTM miRNA Kit (Takara, Japan) and RT Master Mix (Takara, Japan). gDNA was extracted with the A DNA extraction kit (Solarbio, Beijing, China). Briefly, PHCs were collected and resuspended in 500 ml of solution A; 20 μl of RNase A were added, and samples were incubated at 55°C for 10 min. Next, 20 μl of proteinase K were added, and samples were kept in a water bath at 55°C for 30 min. Then, 500 μl of solution B were added, and the solution was thoroughly mixed; 500 μl of absolute ethanol were added and mixed well prior to loading the solution to the adsorption column for 2 min. The solution was centrifuged at 12,000 rpm for 2 min, the supernatant was discarded, and the content of the adsorption column was placed in the collection tube. Next, 600 μl of rinsing solution were added to the adsorption column and centrifuged at 12,000 rpm for 1 min; the waste liquid was discarded, and the column was centrifuged at 12,000 rpm for 2 min. Then, the adsorption column was placed in an incubator at 50°C for 5 min before transferring the adsorption column into a clean centrifuge tube. Finally, 50 μl of eluent were added to the column at room temperature for 5 min and centrifuged at 12,000 rpm for 2 min to obtain the gDNA. qRT‐PCR was performed using SYBR qRT‐PCR Master Mix (Takara) on ABI 7500 Sequencing Detection System (Applied Biosystems, Foster City, CA, USA) following the manufacturer's instructions. Data analysis was performed using QuantStudio™ Real‐Time PCR Software. Convergent and divergent primers for circNFIX and GAPDH were used to amplify circRNA and GAPDH in cDNA and gDNA. The amplification results were analysed by gel electrophoresis and Sanger sequencing. The primers for qRT‐PCR are listed in Table S1.

### 2.5 Western blotting (WB)

The hADSC chondrogenic microspheres and chondrocytes were lysed in radioimmunoprecipitation assay lysis buffer (Cwbio, China). The concentrations of proteins were detected using a bicinchoninic acid kit (Cwbio, China). Protein samples (25 μg) were separated by 10% sodium dodecyl sulphate‐polyacrylamide gel electrophoresis, electrophoresis condition: 80 V 25 min, 120 V 60 min; then protein was transferred onto polyvinylidene fluoride (PVDF) membranes (Millipore, Burlington, MA, USA), transfer membrane condition: 250 mA 120 min. The membrane was blocked with 5% skim milk for 60 min. Membranes were incubated with primary antibodies specific for MMP13 (1:1000, Abcam), Col2a1 (1:1000, Abcam), SOX9 (1:1000, Abcam), GPADH (1:000, affinity), KDM6A (1:1000, Proteintech), H3 (1:1000, Proteintech) and H3K27me3 (1:1000, Proteintech). After incubation with a secondary antibody (1:5000, Cell Signaling Technology, Danvers, MA, USA), the signals were detected using a chemiluminescence kit (P90719, Millipore) and chemiluminescence system (Bio‐Rad, Hercules, CA, USA) and analysed using Image Lab Software.

### 2.6 Immunofluorescence

Chondrocytes were cultured on glass coverslips. After the treatment, cells were fixed with 4% paraformaldehyde for 15 min, permeabilized with 0.1% Triton X‐100 at room temperature for 10 min and then blocked with 1% bovine serum albumin for 30 min. Cells were then incubated with MMP13, COL2A1 and SOX9 (1:200; Abcam) at 4°C overnight. Subsequently, cells were incubated with secondary antibodies conjugated to fluorescent Cy5 dye (1:100; Abcam). The nuclei were stained with 4′,6‐diamidino‐2‐phenylindole dihydrochloride (DAPI) for 10 min at room temperature. Immunofluorescence images were observed under a confocal microscope (Zeiss, Germany).

### 2.7 RNA fluorescent in situ hybridization (FISH)

The probes (human) conjugated to a fluorescent dye for circNFIX and miR‐758‐3p were designed and synthesized by RiboBio (Guangzhou, China). The probes (mouse) for circNFIX were synthesized by Servicebio (Wuhan, China). PHCs were fixed with 4% paraformaldehyde and then permeabilized with 0.1% Triton X‐100 and 10 mM VRC CSK buffer. After dehydration, fixed cells were hybridized at 37°C overnight. For cartilage tissues, sections were deparaffinized, rehydrated and permeabilized by 0.8% pepsin treatment, followed by hybridization at 37°C overnight. After being washed three times, slides were incubated with DAPI for 10 min at room temperature, and the signals were observed using a confocal microscope (Zeiss, Germany).

### 2.8 Pull‐down assay with circNFIX probe

The circNFIX probe was designed by SCbio (Guangzhou, China). SW1353 cells were transfected with circNFIX plasmids. After 24 h, cells were collected and lysed with 1 ml of lysis buffer and irradiated with 254 nm ultraviolet light. The circNFIX probe and Lac Z probes (control probes) mixed with magnetic beads (Life Technologies) were incubated at 25°C for 2 h. Then, the probe‐coated beads were incubated with the cell lysates at 4°C overnight. Non‐specifically bound RNAs were removed by washing, and the remaining RNA was eluted with elution buffer. Finally, total RNA was reverse‐transcribed to complementary DNA (cDNA) and detected by qRT‐PCR.

### 2.9 RNA immunoprecipitation (RIP)

Argonaute2‐RNA immunoprecipitation (Ago2‐RIP) assays were performed using Magna RIPTM RNA‐binding Protein Immunoprecipitation Kit (Millipore) according to the manufacturer's instructions. SW1353 cells were harvested and lysed using RIP lysis buffer. Prepare magnetic beads for immunoprecipitation: pipette 50 μl of magnetic beads into two EP tubes; add 100 μl of diluted anti‐AGO2 antibody (Millipore) or immunoglobulin G (negative control [NC]) (Millipore) to the EP tubes respectively; incubate at room temperature for 30 min. The lysates were then incubated with beads coated with either anti‐AGO2 antibody or IgG antibody at 4°C overnight. The immunoprecipitated RNAs were extracted and reverse‐transcribed into cDNA. CircNFIX and miR758‐3p were detected by qRT‐PCR.

### 2.10 Luciferase reporter assay

Wild type (WT) or mutant circNFIX fragments were inserted into the Xba1 restriction sites of the pSI‐Check2 luciferase vector (Hanbio, Shanghai, China). CircNFIX‐WT and circNFIX‐MUT plasmids, miR‐758‐3p and NC, were transfected into HEK293T cells seeded in 96‐well plates. WT or mutant KDM6A 3′UTR fragments were inserted into the Xba1 restriction sites of the pSI‐Check2 luciferase vector (Hanbio, Shanghai, China). Plasmids of KDM6A 3′UTR‐wt and KDM6A 3′UTR‐mut, miR‐758‐3p and NC were transfected into HEK293T cells seeded in 96‐well plates. After 48 h of incubation, firefly and Renilla luciferase activities were measured using the Promega Dual‐Luciferase system (Promega, USA).

### 2.11 Destabilization of the medial meniscus (DMM) model of OA

All mice were purchased from Jiangsu, China. The experimental animal procedures were approved by the Animal Research Institute (SYSU‐IACUC‐2020‐000270). A total of 45 male, 10‐week‐old WT C57 BL/6 J mice were used for in vivo experiments. A model of OA was established by DMM as described by Li et al.^16^ Mice were subjected to DMM surgery on the left knee, and a sham operation was performed on the right knee. After 4 weeks, mice were randomly divided into three groups (*n* = 15): sham, DMM + vector and DMM + ovcircNFIX. For the DMM + vector group, an adeno‐associated virus (AAV)‐vector (10 μl, 1.3 × 10E11 pfu/ml) was injected into the left knees of the mice; for the DMM + ovcircNFIX group, AAV‐circNFIX (mouse species, mmu_circ_0001704) (10 μl, 2.2 × 10E11 pfu/ml) was injected into the left knees of the mice. The procedure was repeated after 4 weeks. After 12 weeks, mice were sacrificed, and the knee joints were collected. The knee contents were analysed using micro‐CT, WB and immunohistochemistry (IHC).

### 2.12 Micro‐CT analysis

The knees harvested from the animal study were fixed in 4% paraformaldehyde. Then, all joints were scanned using a micro‐CT instrument according to the manufacturer's instructions (Zeiss, Germany); samples were scanned in the instrument tube with 50 kV voltage, 30 W power and 500 μA current for 115 min. Three‐dimensional reconstruction and data analysis were performed using the software (Multimodal 3D Visualization) provided by the manufacturer (Zeiss, Germany). Osteophytes around the joints, including the medial and lateral sides of the tibia and femur, were defined as the region of interest (ROI). ROI was selected from periarticular osteophytes using Mimics 5.0 and marked in red. The ROI size was calculated blinded on all four condyles of the knees (medial and lateral sides of the tibia and femur), and the average was used for statistical analysis. The osteophyte scores were measured according to the description by Altman et al.^22^

### 2.13 Immunohistochemistry

The hADSC microspheres induced to undergo chondrogenesis and cartilage specimens were fixed in 4% paraformaldehyde and paraffin‐embedded. Then, the tissues were cut into 5‐μm sections. For the hADSC microspheres, parts of the sections were stained with Alcian blue; for the cartilage tissues, part of the sections, after deparaffinization and rehydration, were stained with haematoxylin–eosin (HE) and safranin‐O/fast green. The microsphere and cartilage sections were incubated with primary antibodies against COL2A1 and MMP3, respectively, according to a previous protocol.^16^ Three fields of IHC images were selected, and the positive staining of chondrocytes was calculated to obtain a mean value. Cartilage destruction was scored by two observers blinded to group information using the Osteoarthritis Research Society International (OARSI) grading system.^23^

### 2.14 Statistical analysis

Statistical analyses were performed using GraphPad Prism 8 and SPSS v22.0. A Student's *t*‐test or Mann–Whitney *U*‐test was performed to identify differences between the two groups. A one‐way analysis of variance and Kruskal–Wallis tests were used for multiple groups. The data are presented as the mean ± standard deviation (SD). For all analyses, group differences were considered statistically significant at *p* < 0.05.

## 3 RESULTS

### 3.1 Expression pattern of circNFIX during hADSC chondrogenesis and in normal and OA cartilages

**FIGURE 1:**
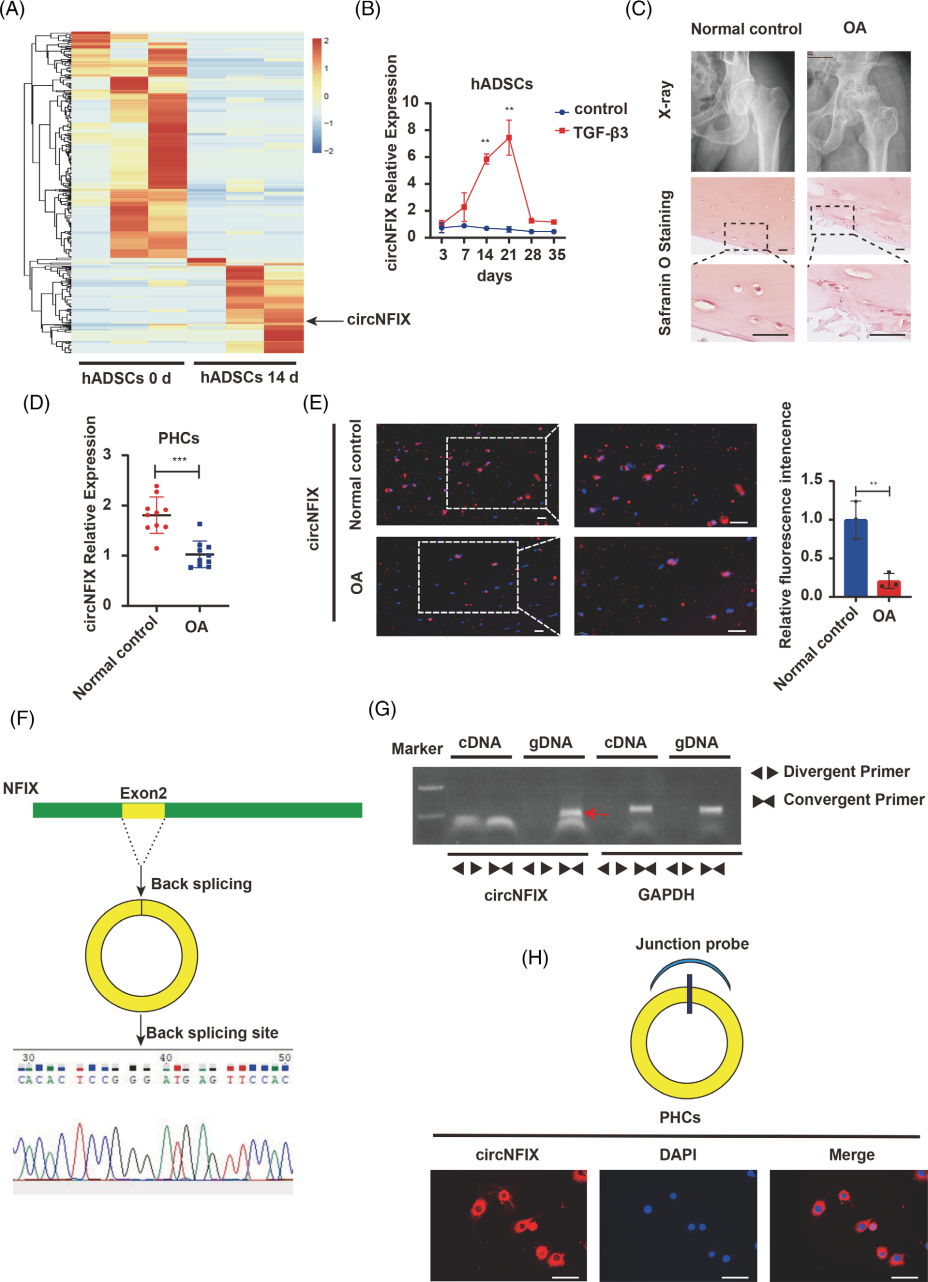
CircNFIX expression pattern during hADSC chondrogenesis and in OA cartilage. (A) Heat map of all differentially expressed circRNAs between the middle stage and early stage of hADSC chondrogenic differentiation. (B) hADSCs were induced to undergo chondrogenesis, and the expression of circNFIX was measured by qRT‐PCR, one‐way ANOVA and Tukey's multiple comparison test. (C) X‐ray and safranin‐O/fast green staining of the cartilage from OA or normal control patients. (D) CircNFIX RNA expression in chondrocytes between OA and normal controls, *n* = 10, data are shown as means ± SDs, Student's *t*‐test. (E): CircNFIX expression in OA cartilage and control cartilage tissues evaluated by FISH, *n* = 3, Student's *t*‐test. (F) CircNFIX is cyclized from NFIX exon 2, the black arrow showed the head‐to‐tail splicing sites of circNFIX. (G) Divergent and convergent primers amplified circNFIX from cDNA and genomic DNA. (H) FISH revealed the localization of circNFIX in chondrocytes; nuclei were stained with DAPI. Scale bar = 50 μm. *p < 0.05, **p < 0.01, ***p < 0.001. All data are shown as means ± SDs of three independent experiments in (A), (B) and (E). DAPI, 4′,6‐diamidino‐2‐phenylindole; FISH, fluorescence in situ hybridization; hADSCs, human adipose‐derived stem cells; OA, osteoarthritis; PHCs, primary human chondrocytes; qRT‐PCR, quantitative reverse transcription‐polymerase chain reaction

hADSCs were induced to undergo chondrogenic differentiation. Microarray analyses were performed to analyse the differential circRNA expression between differentiated samples at 0 and 14 days. We identified 365 upregulated and 213 downregulated circRNAs (Table S2). Among these differentially expressed circRNAs, we found that circNFIX was significantly upregulated during hADSC chondrogenesis on day 14 (Figure 1A). In the early and middle stages of chondrogenic differentiation, circNFIX expression gradually increased and peaked at 21 days after cartilage maturation; in the late stage of chondrogenic differentiation, cartilage degeneration occurred, and circNFIX expression was downregulated (Figure 1B). Furthermore, circNFIX expression was evaluated in cartilage samples collected from OA and normal control (Figure 1C), and the result showed that circNFIX was downregulated in OA samples (Figure 1D,E).

We obtained the circNFIX sequence from circBase and found that circNFIX is circularly spliced from the second exon of NFIX. We first designed convergent and divergent primers to amplify circNFIX using cDNA and genomic DNA (gDNA). Convergent primers could amplify the linear form of the second exon of NFIX and circNFIX in cDNA and gDNA; however, convergent primers could only amplify GAPDH in gDNA but not in cDNA. On the other hand, divergent primers could amplify circNFIX in cDNA but not in gDNA, suggesting that circNFIX is not present in genomic DNA and is a circular splicing product of NFIX mRNA (Figure 1G). The results of the Sanger sequencing analysis of PCR products were in accordance with the circNFIX sequence (Figure 1F). In addition, FISH revealed that circNFIX was located mainly in the cytoplasm of chondrocytes (Figure 1H).

### 3.2 CircNFIX regulates hADSC chondrogenesis and chondrocyte extracellular matrix (ECM) metabolism

**FIGURE 2:**
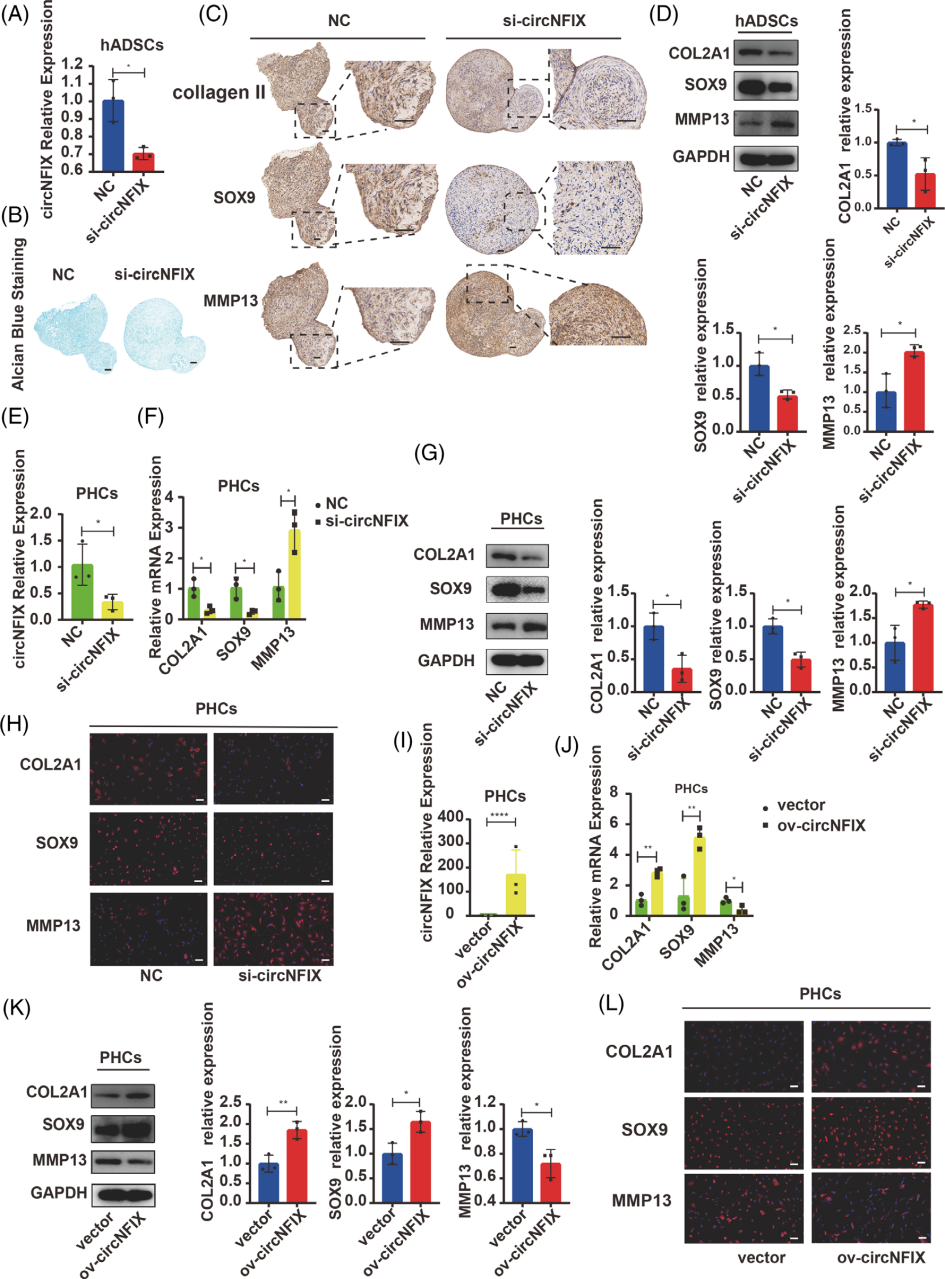
CircNFIX maintained hADSC chondrogenesis and prevented chondrocyte degeneration. hADSCs were transfected with circNFIX siRNA and induced to undergo chondrogenesis for 14 days. (A) CircNFIX expression was measured by qRT‐PCR, Student's *t*‐test. (B) Alcian blue staining was performed on the sections of hADSC chondrogenic microspheres, scale bar = 100 μm. (C, D) The expression of COL2A1, SOX9 and MMP13 in chondrogenic microspheres was estimated by IHC, WB, scale bar = 100 μm, Student's *t*‐test. (E): PHCs were transfected with circNFIX siRNA, circNFIX expression was measured by qRT‐PCR, Student's *t*‐test. (F–H) The expression of COL2A1, SOX9 and MMP13 were estimated by qPCR, WB and IF, Student's *t*‐test. (I) PHCs were transfected with circNFIX plasmids, circNFIX expression was measured by qRT‐PCR, Student's *t*‐test. (J–L) The expression of COL2A1, SOX9 and MMP13 were estimated by qPCR, WB and IF, scale bar = 50 μm, Student's *t*‐test. *p < 0.05, **p < 0.01, ***p < 0.001. All data are shown as means ± SDs of three independent experiments in (A), (D), (E), (F), (G), (I), (J) and (K). hADSCs,human adipose‐derived stem cells; IF, immunofluorescence; IHC, immunohistochemistry; PHCs, primary human chondrocytes; qRT‐PCR, quantitative reverse transcription‐polymerase chain reaction; siRNA, small interfering RNA; WB, western blotting

hADSCs were transfected with circNFIX siRNA and then differentiated into chondrogenic lineage for 14 days. Alcian blue staining revealed that knockdown of circNFIX attenuated hADSC chondrogenesis (Figure 2A,B) with upregulated MMP13 and downregulated COL2A1 and SOX9 expression (Figure 2C,D). PHCs were transfected with siRNA and circNFIX plasmids (Figure 2E,I). The knockdown of circNFIX upregulated MMP13 and downregulated COL2A1 and SOX9 expression (Figure 2F–H). Furthermore, circNFIX overexpression downregulated MMP13 and upregulated COL2A1 and SOX9 (Figure 2J–L). These results suggested that circNFIX regulated hADSC chondrogenesis and mediated the ECM metabolism in PHCs.

### 3.3 CircNFIX acted as a miRNA sponge

**FIGURE 3:**
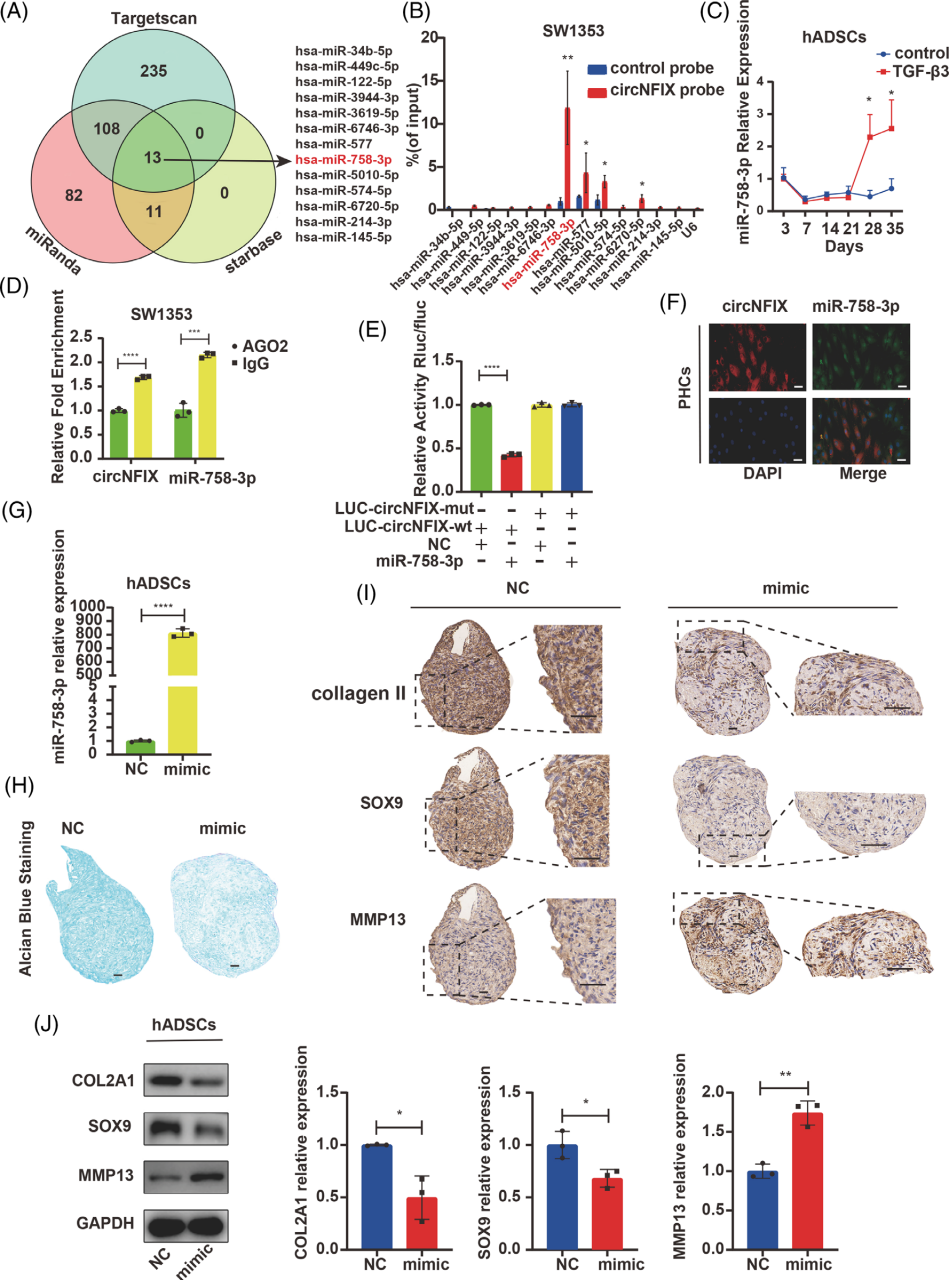
CircNFIX serves as a sponge for miR‐758‐3p in hADSCs chondrogenic differentiation. (A) Schematic illustration to show the overlapping of the target miRNAs of circNFIX, as predicted by Starbase, TargetScan and miRanda. (B) RNA pull‐down assay and the following qRT‐PCR were used to test the miRNA predicted by Starbase, TargetScan and miRanda. Relative levels of circNFIX were normalized to the levels of input versus control (lac Z) probe, Student's *t*‐test. (C) hADSCs were induced to undergo chondrogenesis, and the expression of miR‐758‐3p was measured by qRT‐PCR, one‐way ANOVA and Tukey's multiple comparison test. (D) AgO2‐RIP assay was performed to detect circNFIX and miR758‐3p can bind to Ago2, Student's *t*‐test. (E) HEK‐293T cells were co‐transfected with miR‐758‐3p‐mimic or NC and a luciferase reporter construct containing WT or MUT circNFIX, one‐way ANOVA and Tukey's multiple comparison test. (F) FISH images showed the co‐localization of circNFIX and miR‐758‐3p in PHCs, scale bar = 50 μm. (G) hADSCs were transfected with miR‐758‐3p‐mimic and induced to undergo chondrogenesis for 14 days. CircNFIX expression was measured by qRT‐PCR. (H) Alcian blue staining was performed on the sections of hADSC chondrogenic microspheres, scale bar = 100 μm. (I, J) The expression of COL2A1, SOX9 and MMP13 in chondrogenic microspheres was estimated by IHC, WB, scale bar = 100 μm, Student's *t*‐test. *p < 0.05, **p < 0.01, ***p < 0.001. All data are shown as means ± SDs of three independent experiments in (B), (C), (D), (E), (G) and (J). AGO2, Argonaute2; FISH, fluorescence in situ hybridization; hADSCs, human adipose‐derived stem cells; IF, immunofluorescence; MUT, mutant; NC, negative control; OA, osteoarthritis; PHCs, primary human chondrocytes; qRT‐PCR, quantitative reverse transcription‐polymerase chain reaction; RIP, RNA immunoprecipitation; WB, western blotting; WT, wild type

Given that circNFIX was mainly localized in the cytoplasm, we speculated that circNFIX acted as a sponge for certain miRNAs. To explore the mechanisms underlying the regulation of cartilage homeostasis by circNFIX, three databases (TargetScan, Starbase and miRanda) were used to predict the potential target miRNAs. Finally, 13 miRNAs were selected by overlapping prediction results (Figure 3A). The miRNA expression was detected by qRT‐PCR following the pull‐down assay, and we found that miR‐758‐3p was significantly differentially expressed (Figure 3B). During hADSC chondrogenesis, miR‐758‐3p was downregulated in the early stage and upregulated in the late stage of chondrogenic differentiation (Figure 3C). The Ago2‐RIP verified that both circNFIX and miR‐758‐3p could bind to Ago2 (Figure 3D). The luciferase assay confirmed the circNFIX binding to miR‐758‐3p (Figure 3E). FISH experiments further showed the co‐localization of circNFIX and miR‐758‐3p (Figure 3F). In addition, knockdown of circNFIX in chondrocytes did not alter the expression of miR‐758‐3p, and overexpression of miR‐758‐3p mimic also had no effect on the expression of circNFIX (Figure S1). These results indicated that circNFIX served as a miR‐758‐3p sponge.

**FIGURE 4:**
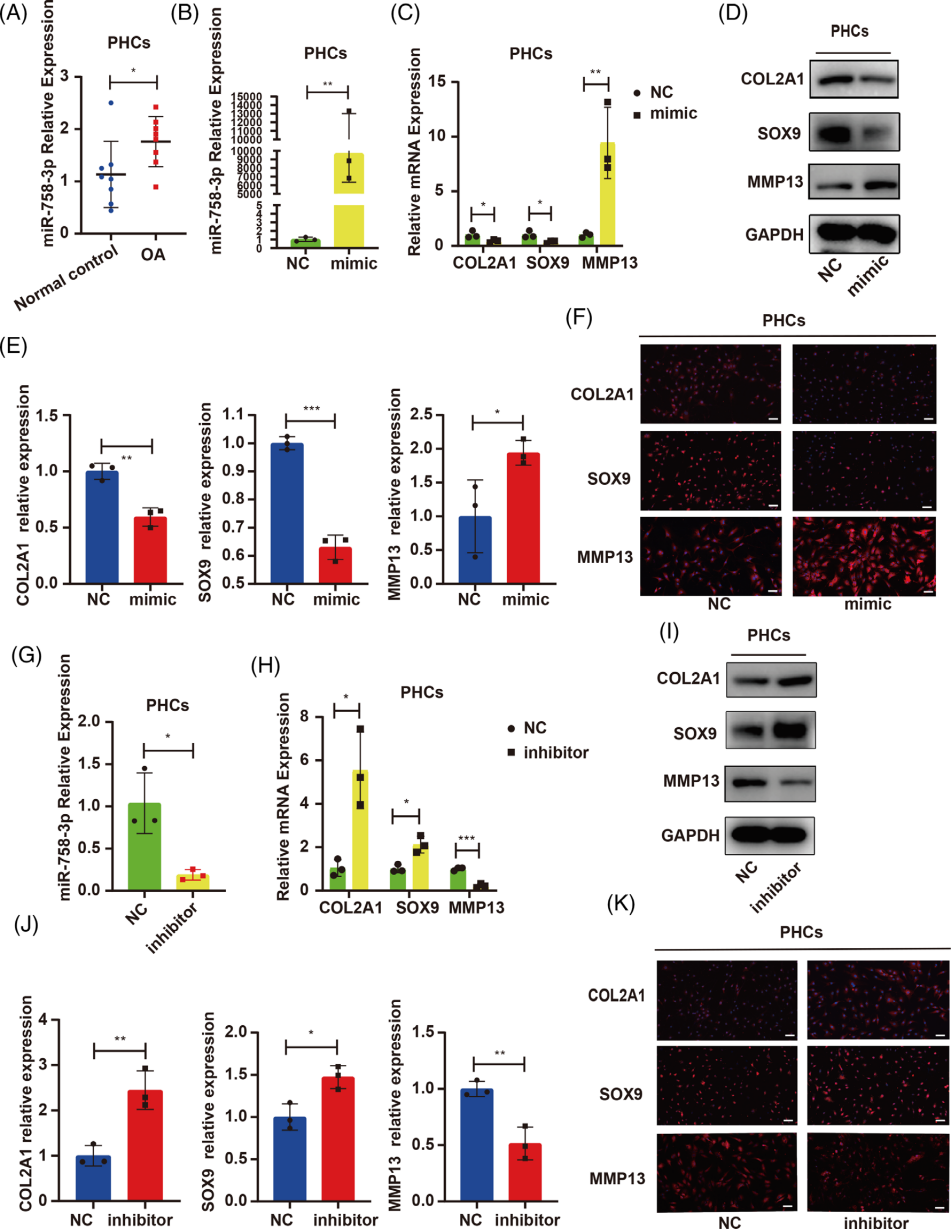
CircNFIX serves as a sponge for miR‐758‐3p in PHCs. (A) The miR‐758‐3p expression in OA and normal control was detected by qRT‐PCR, *n* = 8, data are shown as means ± SDs, Student's *t*‐test. (B) PHCs were transfected with miR‐758‐3p‐mimic, miR‐758‐3p expression was measured by qRT‐PCR, Student's *t*‐test. (C–F) The expression of COL2A1, SOX9 and MMP13 was estimated by qRT‐PCR, WB and IF, Student's *t*‐test. (G) PHCs were transfected with miR‐758‐3p‐inhibitor, miR‐758‐3p expression was measured by qRT‐PCR, Student's *t*‐test. (H–K) The expression of COL2A1, SOX9 and MMP13 was estimated by qRT‐PCR, WB and IF, scale bar = 50 μm, Student's *t*‐test. *p < 0.05, **p < 0.01, ***p < 0.001. All data are shown as means ± SDs of three independent experiments in (B), (C), (D), (E), (G), (H), (I) and (J). IF, immunofluorescence; NC, negative control; OA, osteoarthritis; PHCs, primary human chondrocytes; qRT‐PCR, quantitative reverse transcriptase polymerase chain reaction; WB, western blotting

hADSCs were transfected with miR‐758‐3p‐mimic and differentiated into chondrogenic lineage for 14 days (Figure 3G). We observed that miR‐758‐3p could impair hADSC chondrogenesis (Figure 3H) with upregulated MMP13 and downregulated COL2A1 and SOX9 expression (Figure 3I,J). We also found that miR‐758‐3p was upregulated in PHCs of OA compared with PHCs of normal control (Figure 4A). PHCs were transfected with miR‐758‐3p‐mimic and ‐inhibitor to investigate the effect of miR‐758‐3p (Figure 4B,G). The miR‐758‐3p‐mimic upregulated MMP13 and downregulated COL2A1 and SOX9 expression (Figure 4C,F). Conversely, the inhibition of miR‐758‐3p showed the opposite effects (Figure 4H–K).

### 3.4 CircNFIX mediated hADSC chondrogenesis and PHC homeostasis by interacting with miR‐758‐3p

**FIGURE 5:**
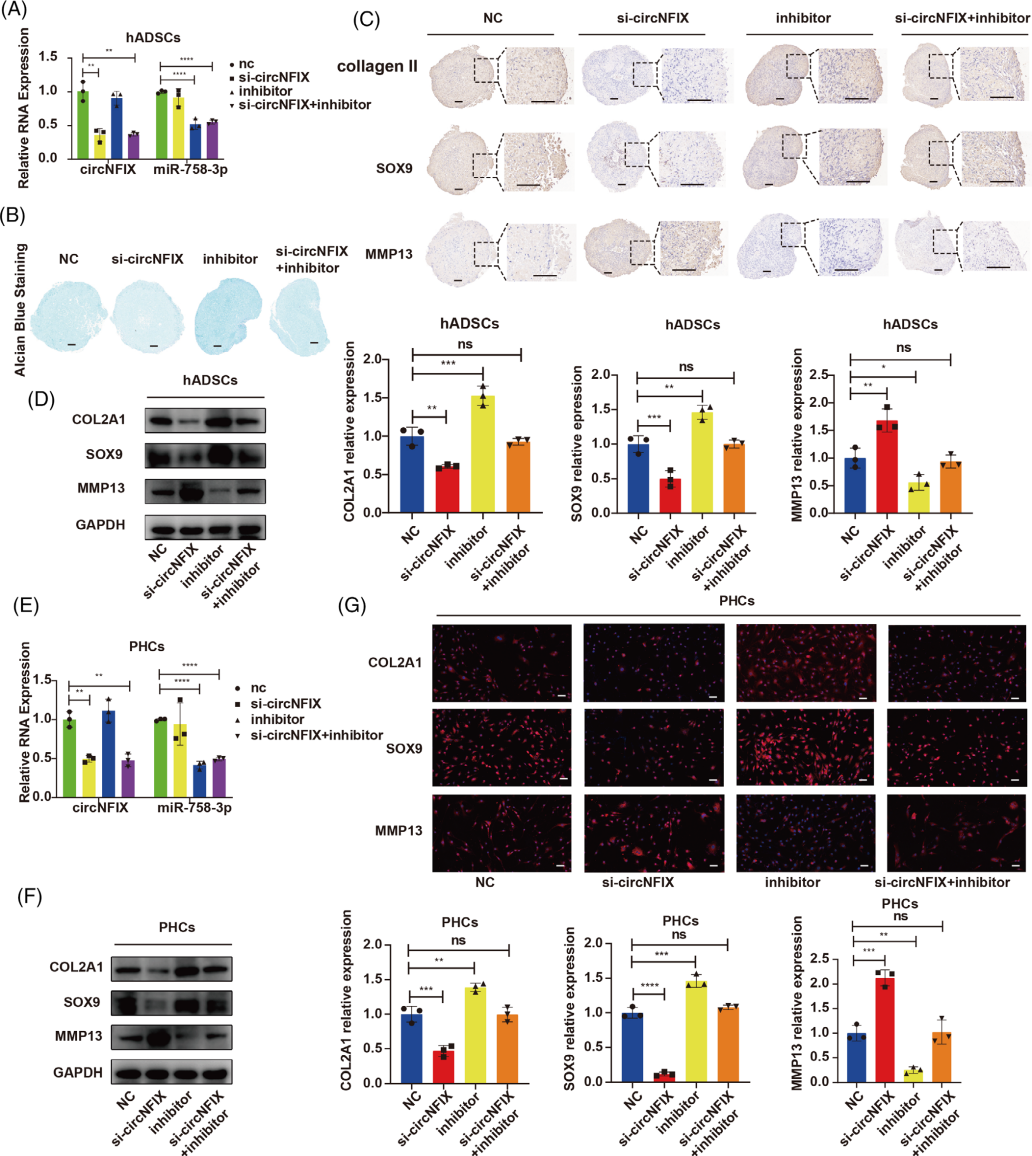
CircNFIX regulates hADSC chondrogenesis and PHC homeostasis by targeting miR‐758‐3p. (A) hADSCs were transfected with circNFIX siRNA or co‐transfected with miR‐758‐3p‐inhibitor and induced to undergo chondrogenesis 14 days, qRT‐PCR was used to detect the expression of circNFIX and miR‐758‐3p, one‐way ANOVA and Tukey's multiple comparison test. (B) Alcian blue staining was performed on the sections of hADSC chondrogenic microspheres, scale bar = 100 μm. (C, D) The expression of COL2A1, SOX9 and MMP13 in chondrogenic microspheres was estimated by IHC and WB, scale bar = 100 μm, one‐way ANOVA and Tukey's multiple comparison test. (E) PHCs were transfected with circNFIX siRNA or co‐transfected with miR‐758‐3p‐inhibitor, qRT‐PCR was used to detect the expression of circNFIX and miR‐758‐3p, one‐way ANOVA and Tukey's multiple comparison test. (F, G) WB and IF were used to detect the expression of COL2A1, SOX9, MMP13, scale bar = 50 μm, one‐way ANOVA and Tukey's multiple comparison test. *p < 0.05, **p < 0.01, ***p < 0.001. All data are shown as means ± SDs of three independent experiments in (A), (D), (E) and (F). hADSCs, human adipose‐derived stem cells; IF, immunofluorescence; PHCs, primary human chondrocytes; qRT‐PCR, quantitative reverse transcriptase polymerase chain reaction; siRNA, small interfering RNA; WB, western blotting

To determine whether circNFIX functions in hADSCs and PHCs by targeting miR‐758‐3p, we designed a rescue experiment. hADSCs were co‐transfected with circNFIX siRNA and miR‐758‐3p‐inhibitor and then induced to undergo chondrogenic differentiation for 14 days. Knockdown of circNFIX alone inhibited hADSC chondrogenesis, with downregulated COL2A1 and SOX9 and upregulated MMP13 expression, while inhibition of miR‐758‐3p promoted chondrogenesis. However, when circNFIX was knocked down and miR‐758‐3p was inhibited simultaneously, the inhibition of miR‐758‐3p could antagonize the effect of circNFIX on chondrogenic differentiation (Figure 5A–D). In PHCs, circNFIX knockdown promoted the catabolism of ECM, with upregulated MMP13 and downregulated COL2A1 and SOX9 expression, while miR‐758‐3p‐inhibitor promoted ECM synthesis. Whereas these effects were rescued by inhibiting both circNFIX and miR‐758‐3p (Figure 5E–G). These results indicated that circNFIX functioned in chondrogenesis and cartilage homeostasis through its interaction with miR‐758‐3p.

### 3.5 miR‐758‐3p and lysine‐specific demethylase6A (KDM6A) binding affects hADSC chondrogenesis and PHC homeostasis

**FIGURE 6:**
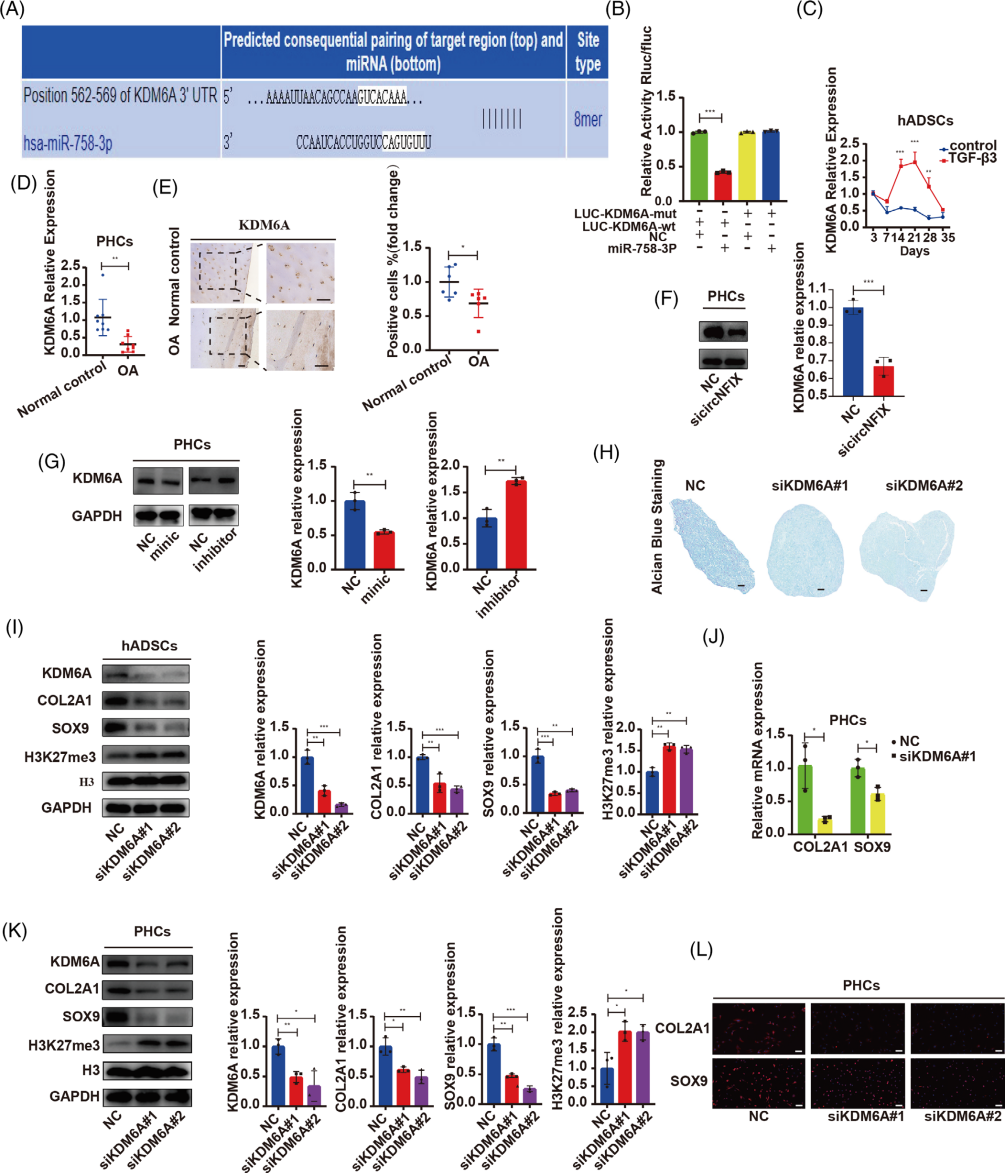
miR‐758‐3p directly targeted KDM6A regulating hADSC chondrogenesis and inducing PHC degeneration. (A) KDM6A was predicted as the direct target of miR‐758‐3p using the Targetscan database. (B) HEK‐293T cells were co‐transfected with miR‐758‐3p‐mimic or NC and luciferase reporter constructs containing WT or MUT 3′‐UTR of KDM6A, *n* = 3, one‐way ANOVA and Tukey's multiple comparison test. (C) hADSCs were induced to undergo chondrogenesis, and the expression of KDM6A was measured by qRT‐PCR, *n* = 3, one‐way ANOVA and Tukey's multiple comparison test. (D) The KDM6A expression in OA and normal control was detected by qRT‐PCR, *n* = 8, data are shown as means ± SDs, Student's test. (E) IHC was used to detect the expression of KDM6A between OA and normal tissues, *n* = 6, data are shown as means ± SDs, scale bar = 100 μm, Student's test. (F) PHCs were transfected with circNFIX siRNA, the expression of KDM6A was measured by WB, Student's test. (G) PHCs were transfected with miR‐758‐3p‐mimic or ‐inhibitor, the expression of KDM6A was measured by WB, Student's test. (H) hADSCs were transfected with KDM6A siRNA and induced to undergo chondrogenesis for 14 days. Alcian blue staining was performed on the sections of hADSC chondrogenic microspheres, scale bar = 100 μm. (I) The expression of COL2A1, SOX9, H3K27me3, H3 and KDM6A in chondrogenic microspheres was estimated by WB, one‐way ANOVA and Tukey's multiple comparison test. (J–L) PHCs were transfected with siRNA of KDM6A, WB was used to detect the expression of COL2A1, SOX9, H3K27me3, H3, and KDM6A, one‐way ANOVA and Tukey's multiple comparison test; qRT‐PCR and IF was used to detect the expression of COL2A1 and SOX9, scale bar = 50 μm, Student's test. *p < 0.05, **p < 0.01, ***p < 0.001. All data are shown as means ± SDs of three independent experiments in (B), (C), (F), (G), (I), (J) and (K). hADSCs, human adipose‐derived stem cells; IF, immunofluorescence; IHC, immunohistochemistry; MUT, mutant; NC, negative control; OA, osteoarthritis; PHCs, primary human chondrocytes; qRT‐PCR, quantitative reverse transcriptase polymerase chain reaction; siRNA, small interfering RNA; WB, western blotting; WT, wild type

We used the TargetScan database to predict the target gene of miR‐758‐3p and found that the 3′‐UTR of human KDM6A contains a potential miR‐758‐3p binding site (Figure 6A). The luciferase assay confirmed the binding of miR‐758‐3p to KDM6A (Figure 6B). KDM6A, a histone H3K27‐specific demethylase also referred to as UTX, was located on Xp11.3.^24^ KDM6A promotes chondrogenic differentiation and prevents OA by demethylating SOX9.^25^ KDM6A was upregulated during the early and middle stages of hADSC chondrogenesis and remarkably downregulated in the late stage (Figure 6C). KDM6A was expressed at low levels in OA samples (Figure 6D,E). In PHCs, circNFIX knockdown inhibited KDM6A expression (Figure 6F). Furthermore, the miR‐758‐3p‐mimic downregulated KDM6A, and the miR‐758‐3p‐inhibitor upregulated KDM6A expression (Figure 6G). hADSCs were transfected with siRNA against KDM6A and then induced to undergo chondrogenic differentiation for 14 days. KDM6A inhibition impaired hADSC chondrogenesis (Figure 6H) and downregulated COL2A1 and SOX9 expression (Figure 6I). In PHCs, KDM6A knockdown remarkably downregulated COL2A1 and SOX9 (Figure 6J–L). We observed that H3K27me3 was upregulated and SOX9 was downregulated after KDM6A inhibition both in hADSC chondrogenic microspheres and PHCs (Figure 6I,K), indicating that the SOX9 was methylated by KDM6A.

**FIGURE 7:**
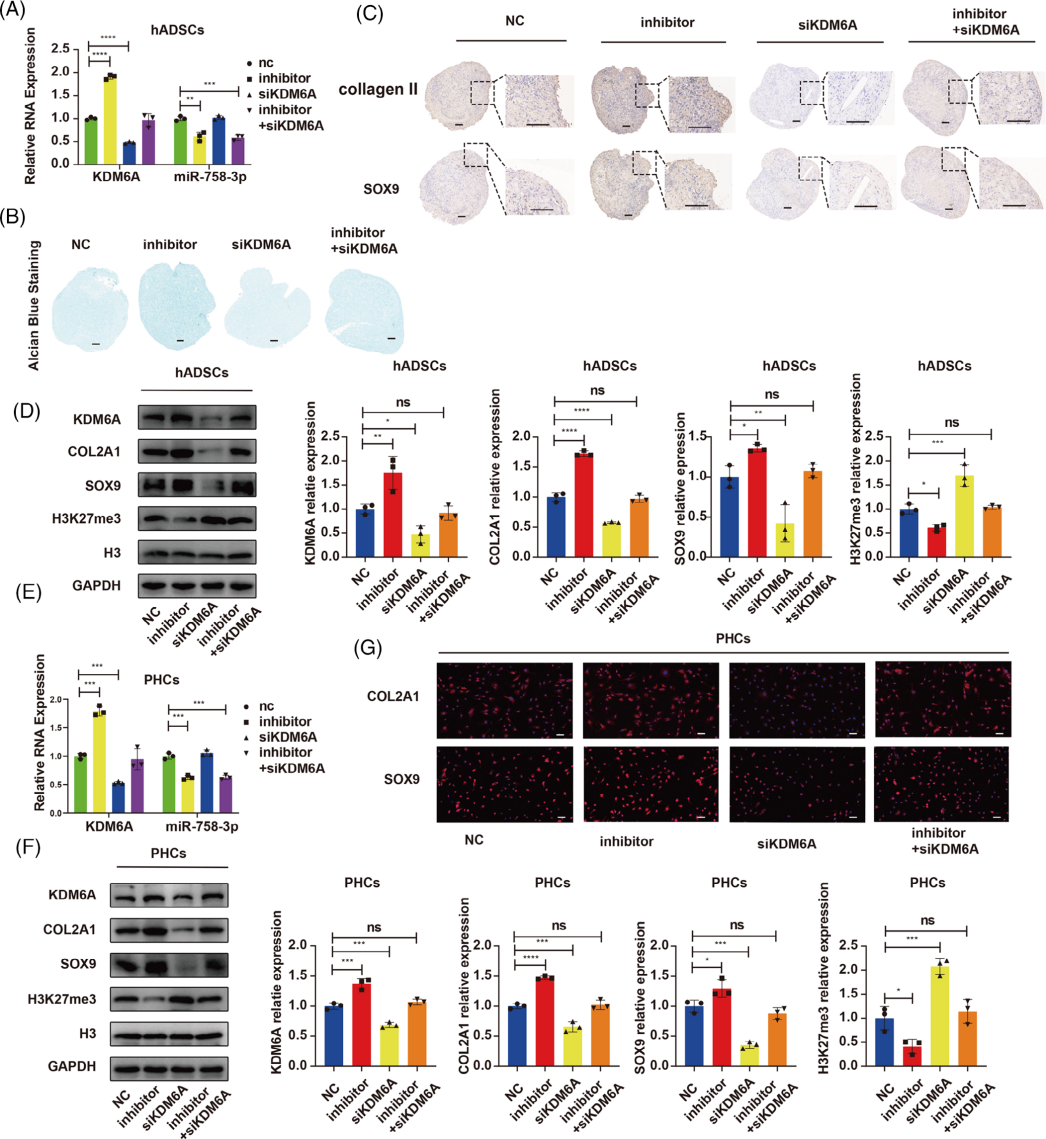
miR‐758‐3p mediated hADSC chondrogenesis and induced PHC degeneration through KDM6A. (A) hADSCs were transfected with miR‐758‐3p‐inhibitor or co‐transfected with KDM6A siRNA and induced to undergo chondrogenesis for 14 days, qRT‐PCR was used to detect the expression of miR‐758‐3p and KDM6A, one‐way ANOVA and Tukey's multiple comparison test. (B) Alcian blue staining was performed on the sections of hADSC chondrogenic microspheres, scale bar = 100 μm. (C) The expression of COL2A1 and SOX9 in chondrogenic microspheres was estimated by IHC, scale bar = 100 μm. (D) WB was used to detect the expression of COL2A1, SOX9, H3K27me3, H3, and KDM6A, one‐way ANOVA and Tukey's multiple comparison test. (E) PHCs were transfected with miR‐758‐3p‐inhibitor alone or co‐transfected with KDM6A siRNA, qRT‐PCR was used to detect the expression of miR‐758‐3p and KDM6A, one‐way ANOVA and Tukey's multiple comparison test. (D) WB was used to detect the expression of COL2A1, SOX9, H3K27me3, H3, and KDM6A, one‐way ANOVA and Tukey's multiple comparison test. (E) IF was used to detect the expression of COL2A1 and SOX9, scale bar = 50 μm. *p < 0.05, **p < 0.01, ***p < 0.001. All data are shown as means ± SDs of three independent experiments in (A), (D), (E) and (F). hADSCs, human adipose‐derived stem cells; IF, immunofluorescence; IHC, immunohistochemistry; PHCs, primary human chondrocytes; qRT‐PCR, quantitative reverse transcriptase polymerase chain reaction; siRNA, small interfering RNA; WB, western blotting

We then designed a rescue experiment to verify whether the effects of miR‐758‐3p expression on cartilage homeostasis were achieved through the KDM6A/SOX9 axis. The miR‐758‐3p‐inhibitor promoted hADSC chondrogenesis and upregulated COL2A1 and SOX9 expression, while siKDM6A inhibited chondrogenesis. However, the effect of miR‐758‐3p‐inhibitor was blocked by knockdown of KDM6A (Figure 7A–D). In PHCs, the inhibition of miR‐758‐3p upregulated COL2A1 and SOX9, and these effects were abolished when co‐transfected with miR‐758‐3p‐inhibitor and siRNA of KDM6A (Figure 7E–G). We observed that KDM6A was upregulated and H3K27me3 was downregulated in both hADSCs and PHCs when miR‐758‐3p was inhibited, whereas KDM6A knockdown restored these effects (Figure 7D,F). These results suggested that miR‐758‐3p inhibition upregulated KDM6A and promoted SOX9 demethylation, as indicated by H3K27me3 downregulation. Taken together, our results revealed that miR‐758‐3p attenuated hADSC chondrogenesis and induced PHC degeneration via the KDM6A/SOX9 axis.

### 3.6 Injection of circNFIX prevented OA progression in the DMM model

**FIGURE 8:**
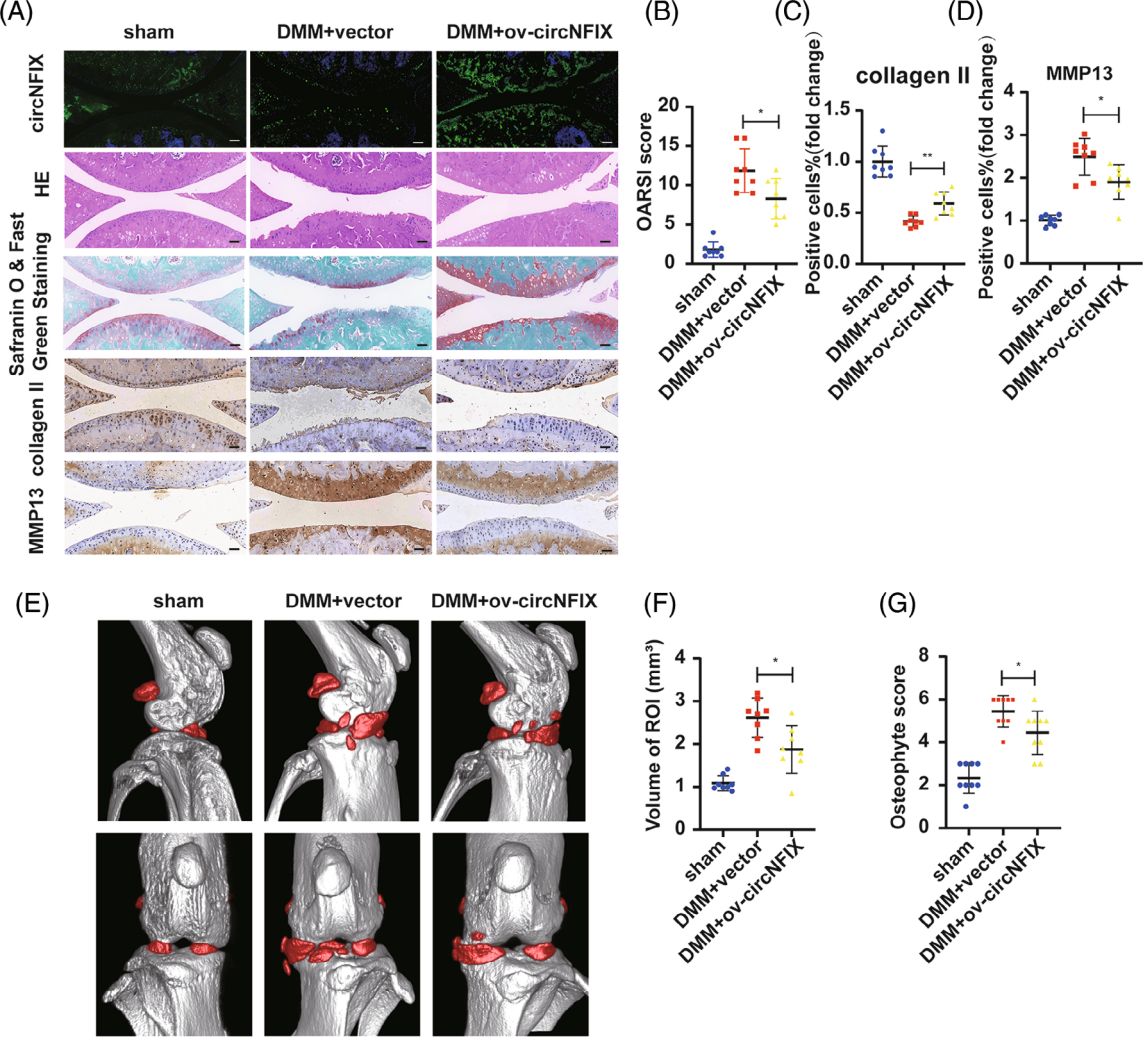
CircNFIX alleviated OA progression in vivo. (A) FISH for circNFIX, HE and Safranin‐O/fast green staining, IHC staining for COL2A1 and MMP13 of the cartilage from the experimented mice joints. (B) The staining results were measured by the OARSI scoring system, *n* = 8, one‐way ANOVA and Tukey's multiple comparison test. (C, D) Quantitative analysis of the IHC staining results, one‐way ANOVA and Tukey's multiple comparison test. (E) Micro‐CT scan and three‐dimensional reconstruction of joints from the experimented mice. (F, G) Quantitative analysis of the volume of the ROI and osteophytes score. The ROI is marked in red for periarticular osteophytes; *n* = 8, one‐way ANOVA and Tukey's multiple comparison test. All data are shown as means ± SDs. Scale bar = 100 μm. *p < 0.05, **p < 0.01, ***p < 0.001. FISH, fluorescence in situ hybridization; HE, haematoxylin–eosin; IHC, immunohistochemistry; micro‐CT, micro‐computed tomography; OARSI, Osteoarthritis Research Society International; ROI, region of interest

To further investigate the functions of circNFIX in vivo, circNFIX‐AAV was intra‐articularly administered to DMM mice. HE, Safranin‐O and fast green staining showed cartilage degeneration in the DMM model. However, the effects were alleviated after AAV‐circNFIX injection relative to the control (Figure 8A). Quantitative analysis with OARSI scoring showed that AAV‐circNFIX significantly lowered OARSI scores (Figure 8B). IHC revealed that the injection of AAV‐circNFIX could restore MM13 expression and increase COL2A1 expression compared with that of the DMM model (Figure 8C,D). We performed micro‐CT analysis and three‐dimensional reconstruction of the knee joints to evaluate osteophytosis. The AAV‐circNFIX injection alleviated osteophytes with significantly decreased osteophyte volume and scores compared with those of the control (Figure 8E–G). Altogether, these results suggested that circNFIX overexpression prevented OA progression in a DMM murine model.

## 4 DISCUSSION

OA is the most common joint disorder, affecting 37% of people aged over 60.^2^ The aetiology of OA involves mechanical, inflammatory and metabolic factors.^26^ No drugs are available to repair damaged cartilage in OA and maintain joint homeostasis. Currently, stem cell composite materials have emerged as a promising strategy to repair cartilage in OA treatment. However, the main issues curtailing the clinical application of composite material, maintaining the cartilage properties and preventing cartilage degeneration, remain resolved.

Several studies have reported that circRNAs regulate ECM degradation, inflammation and apoptosis.^27^ CircRNA can enhance MSC chondrogenesis and prevent cartilage degeneration.^13^ CircPDE4B protects against articular cartilage degeneration by acting as a scaffold for RIC8A and MID1.^28^ In the present study, we found that circNFIX was overexpressed in the early and middle stages and downregulated in the late stage of chondrogenic differentiation. Moreover, circNFIX was downregulated in OA cartilage. Gain‐of‐function and loss‐of‐function approaches indicated that circNFIX could promote hADSC chondrogenesis and prevent cartilage degeneration in vitro and in vivo. These results indicated that circNFIX could be used to preserve cartilage differentiation and cartilage homeostasis.

A previous study reported that circRNA acts as a bait for miRNA, a scaffold for circRNA‐protein complexes, or a translation template for regulating gene expression.^29^ However, most recent studies have focused on the function of circRNAs as miRNA sponges. Here, we discovered that circNFIX was mainly located in the cytoplasm. Further, the Ago2‐RIP assay results indicated that circNFIX could bind to Ago2, suggesting that circNFIX may serve as a miRNA sponge. According to three databases (TargetScan, Starbase and miRanda), qRT‐PCR analysis following RNA‐pull‐down, FISH and luciferase assays, we found that miR‐758‐3p can interact with circNFIX. Evidence has shown that miRNAs are associated with chondrogenesis and cartilage homeostasis. MiR‐23a‐3p attenuates hADSC chondrogenesis via targeting SOX6/SOX5.^30^ MiR‐455‐3p prevents chondrogenic differentiation through the modification of DNA methylation.^31^ In the present study, we found that miR‐758‐3p could impair hADSC chondrogenesis and induce PHC degeneration. These results suggested that miR‐758‐3p may target cartilage‐specific genes. Further, the assay results indicated that miR‐758‐3p interacted with KDM6A and subsequently suppressed its expression.

KDM6A is a histone H3K27‐specific demethylase linked to homeotic gene expression and cellular reprogramming.^32^ Studies have shown that KDM6A plays a critical role in embryonic stem cell (ESC) differentiation, tissue‐specific development and tumorigenesis.^33^ In the present study, we found that KDM6A was upregulated in the early and middle stages and downregulated in the late stage of hADSC chondrogenesis. Moreover, IHC showed that KDM6A expression was low in OA samples. These results indicated that KDM6A was associated with chondrogenesis and cartilage homeostasis. A further experiment showed that KDM6A knockdown attenuated hADSC chondrogenesis and promoted PHCs degeneration. However, the effects of KDM6A inhibition were rescued by a miR‐758‐3p‐inhibitor.

According to a previous study, KDM6A regulates the differentiation and formation of induced pluripotent stem cells (iPSC) by mediating histone demethylation.^34^ KDM6A reportedly promotes the chondrogenic differentiation of periodontal ligament stem cells via SOX9 demethylation.^35^ We speculated that KDM6A‐mediated hADSC chondrogenic differentiation and cartilage homeostasis was related to its demethylation activity, especially promoting SOX9 demethylation. SOX9 is a transcription factor of COL2A1 and is essential for chondrocyte differentiation and cartilage formation.^36^ In our study, KDM6A knockdown suppressed COL2A1 and SOX9 expression in PHCs and hADSCs after 14 days of induced chondrogenic differentiation. H3K27me3 is a marker of methylation levels. We found that H3K27me3 was upregulated, corresponding to SOX9 downregulation after KDM6A inhibition. In addition, miR‐758‐3p inhibitor promoted KDM6A expression and increased SOX9 levels; H3K27me3 was correspondingly downregulated. These findings indicated that KDM6A regulated SOX9 methylation. Therefore, these results suggested that KDM6A mediated hADSC chondrogenesis and cartilage homeostasis via SOX9 demethylation.

The study revealed that circNFIX mediated chondrogenesis and cartilage homeostasis, and its underlying mechanism was through sponging downstream miR‐758‐3p/KDM6A. However, the limitations are that our sample size was relatively small, and we did not rule out other potential mechanisms involving circNFIX. Further studies should include larger OA cohorts and explore additional mechanisms.

## 5 CONCLUSIONS

We unveiled that circNFIX regulated chondrogenesis and cartilage homeostasis by targeting the miR758‐3p/KDM6A axis. The circNFIX/miR‐758‐3p/KDM6A axis may be an effective therapeutic target for cartilage degeneration and OA treatment.

## AUTHOR CONTRIBUTIONS

Ziji Zhang and Yan Kang were responsible for the study concept and design; Hongyi Liao, Qingqiang Tu and Yunze Kang performed the experiments, conducted data analysis and wrote the manuscript; Yuanyuan Xu reviewed and revised the manuscript; Guping Mao, Zhiwen Li and Puyi Sheng provided cartilage tissue specimens; Xudong Wang, Yiyang Xu, Dianbo Long and Shu Hu acquired the data.

## FUNDING INFORMATION

This study was supported by the National Natural Science Foundation of China (grant number: 81874014) and The Natural Science Foundation of Guangdong Province, China (grant number: 2022A1515012279).

## CONFLICT OF INTEREST

The authors declare that they have no conflict of interest.

## Supporting information


**Table S1** Primers used for qRT‐PCR.

**Table S2** circRNA expression between hADSCs chondrogenesis 0‐day and 14‐day samples.

**Figure S1** CircNFIX and miR‐758‐3p cannot affect each other's expression. (a) PHCs were transfected with circNFIX siRNA, qRT‐PCR was used to detect the expression of circNFIX and miR‐758‐3p, student's test. (b) PHCs were transfected with miR‐758‐3p‐mimic, qRT‐PCR was used to detect the expression of circNFIX and miR‐758‐3p, student's test. *p < 0.05, **p < 0.01, ***p < 0.001. All data are shown as means ± SDs of three independent experiments. qRT‐PCR, quantitative reverse transcriptase polymerase chain reaction.

## Data Availability

The datasets generated during the current study are available on the NCBI website (accession number: PRJNA755468; https://www.ncbi.nlm.nih.gov/search/all/?term=PRJNA755468).
