# Supplementary figures and images for: CircNFIX regulates chondrogenesis and cartilage homeostasis by targeting the miR758‐3p/KDM6A axis
Source: Cell Prolif. 2022 Jul 5;55(11):e13302. doi: 10.1111/cpr.13302 (PMC9628241; doi:10.1111/cpr.13302)

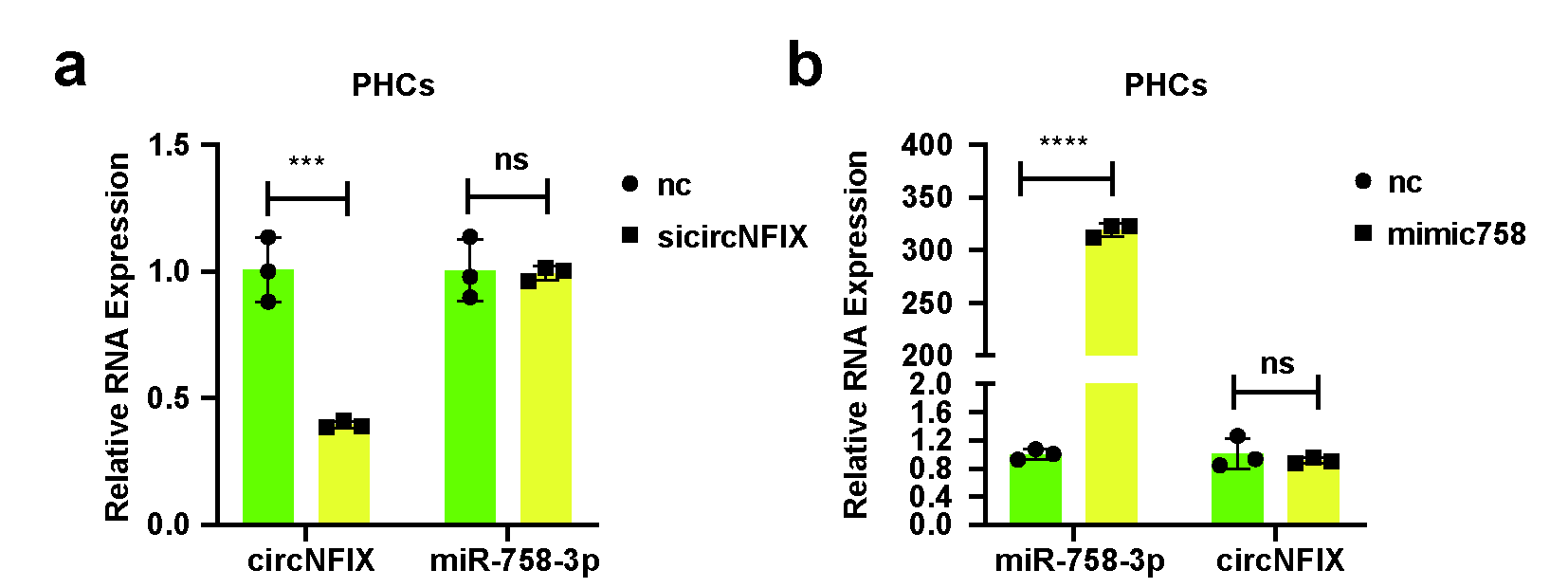

Supplement: Supplementary file 3 — Figure S1 CircNFIX and miR‐758‐3p cannot affect each other's expression. (a) PHCs were transfected with circNFIX siRNA, qRT‐PCR was used to detect the expression of circNFIX and miR‐758‐3p, student's test. (b) PHCs were transfected with miR‐758‐3p‐mimic, qRT‐PCR was used to detect the expression of circNFIX and miR‐758‐3p, student's test. *p < 0.05, **p < 0.01, ***p < 0.001. All data are shown as means ± SDs of three independent experiments. qRT‐PCR, quantitative reverse transcriptase polymerase chain reaction. [file CPR-55-e13302-s001.tif]
